# Cytomegalovirus Endotheliitis After Penetrating Keratoplasty

**DOI:** 10.4274/tjo.galenos.2020.47568

**Published:** 2020-10-30

**Authors:** Tuna Çelik Büyüktepe, Nilüfer Yalçındağ

**Affiliations:** 1 Dr. Nafiz Körez Sincan State Hospital, Clinic of Ophthalmology, Ankara, Turkey; 2 Ankara University Faculty of Medicine, Department of Ophthalmology, Ankara, Turkey

**Keywords:** Penetrating keratoplasty, cytomegalovirus, viral endotheliitis, graft rejection

## Abstract

Cytomegalovirus (CMV)-related corneal endotheliitis is an inflammation of the corneal endothelium caused by CMV. It may occur de novo or after ocular surgery in otherwise healthy individuals. In patients who have undergone keratoplasty, the differential diagnosis of viral endotheliitis and immune-related graft rejection is challenging due to the similar clinical findings. Here we report a patient who underwent penetrating keratoplasty and was using local and systemic immunosuppressive agents due to previous history of graft rejection. At postoperative year 4, ophthalmologic examination revealed localized corneal edema, coin-shaped keratic precipitates, and increased intraocular pressure, consistent with viral endotheliitis. Polymerase chain reaction revealed CMV-DNA amplification in the aqueous humor sample. Valganciclovir treatment was started and the symptoms improved in 2 months. It should be kept in mind that local or systemic immunosuppressants used after keratoplasty may trigger CMV reactivation. Anti-CMV treatment should be initiated immediately in patients with coin-shaped keratic precipitates.

## Introduction

Viral endotheliitis is endothelial inflammation and damage characterized by corneal edema, keratic precipitates (KPs), mild anterior chamber reaction, and elevated intraocular pressure (IOP).^1^ The main causative agents are herpes simplex virus (HSV), varicella zoster virus (VZV), mumps virus, and cytomegalovirus (CMV).^2^

CMV-associated endotheliitis can occur de novo or secondary to ocular surgery in immunocompetent individuals.^3^ Although KPs with typical location and appearance (coin-shaped) facilitate the differential diagnosis, it is mainly confirmed by isolation of viral antibodies or DNA in the aqueous humor by polymerase chain reaction (PCR).^4^ CMV endotheliitis after keratoplasty may be misdiagnosed as graft rejection; therefore, deciding on the treatment protocol can be challenging. Corneal vascularization accompanying stromal edema, different KP pattern, and quick response to steroid therapy are clinical signs of immune-related graft rejection.^5^

This report describes a case of CMV endotheliitis that occurred at postoperative 4 years after partial penetrating keratoplasty (PPK) in a patient with a history of graft rejection and under immunosuppressive therapy. The aim is to provide information to differentiate two clinical entities which are frequently confused and raise our colleagues’ awareness of CMV endotheliitis.

## Case Report

**Figure 1:**
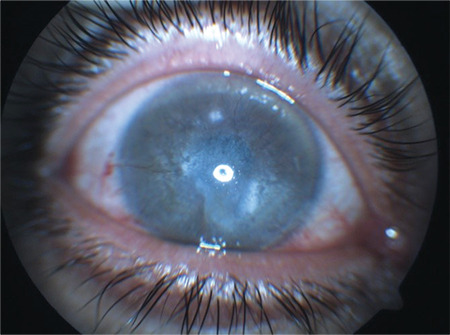
An anterior segment photograph of the patient before keratoplasty shows vernal keratoconjunctivitis scar

A 42-year-old man presented to our hospital with complaint of low vision in both eyes. His past medical history revealed severe ocular itching and redness in childhood suggesting ocular allergy, and his vision had declined over the last 12 years. On ophthalmological examination, his best corrected visual acuity (BCVA) was counting fingers from 2 meters in the right eye and 0.2 in the left eye. On anterior segment examination, total corneal opacity was observed in the right eye (Figure 1) and near total opacity in the left eye. Intraocular pressure (IOP) was 15 mmHg in the right eye and 14 mmHg in the left eye. Because posterior segment evaluation of the right eye was limited by dense corneal opacity, ocular ultrasonography was performed and revealed no pathology. Fundoscopy of the left eye was normal.

**Figure 2:**
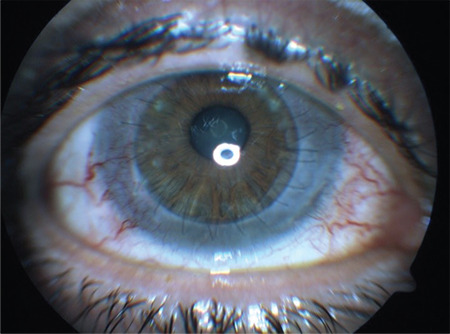
The graft is clear in the early period after partial penetrating keratoplasty

The patient was diagnosed as having vernal keratoconjunctivitis-associated corneal scar and underwent PPK in the right eye. After uncomplicated PPK surgery, the patient received topical ofloxacin drops 5 times a day, dexamethasone drops 5 times a day, chloramphenicol ointment twice a day, and 200 mg oral cyclosporine-A. In the early postoperative period, the graft was clear, BCVA was 0.5, and IOP was 14 mmHg (Figure 2). The corneal sutures were removed at postoperative 12 months. Decreased vision (0.4), graft edema, and medium-sized KP in the graft center were first observed at 18-month follow-up. Suspecting immune-mediated graft rejection, the topical steroid dose was increased and intravenous pulse methylprednisolone (250 mg 4 times a day for 3 days) was added to the current immunosuppressant treatment. At 1 week after pulse steroid therapy, the KPs dissappeared, graft edema resolved, and BCVA increased to 0.5.

**Figure 3:**
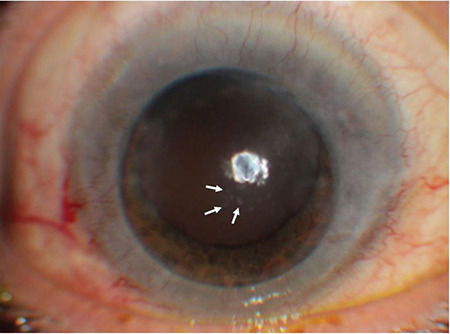
At postoperative 4 years, the patient under ongoing local and systemic immunosuppressive therapy developed coin-shaped keratic precipitates (arrows) in the central cornea and edema in the surrounding stroma

At postoperative year 4, the cyclosporine therapy was discontinued gradually. Three months later, his BCVA had declined again to 0.4. On anterior segment examination, coin-shaped KPs and corneal edema were observed in the paracentral area of the graft cornea (Figure 3). Additionally, mild anterior chamber reaction (+1) was present and IOP was elevated to 30 mmHg. Viral endotheliitis was suspected and etiological agents were investigated by performing anterior chamber paracentesis and PCR analysis. CMV-DNA was isolated in the aqueous humor. The patient was started on oral valganciclovir 900 mg twice a day, topical dexamethasone drops 5 times a day, ganciclovir gel 5 times a day, and topical dorzolamide-timolol drop twice a day. After 2 months of follow-up, graft edema and anterior chamber reaction had resolved and IOP was under control with topical antiglaucomatous treatment.

## Discussion

Ocular CMV infection, especially retinitis, occurs in patients with a suppressed T-cell-related immune response due to acquired immunodeficiency syndrome (AIDS) or organ transplantation.^6^ On the other hand, in immunocompetent patients, mainly the anterior segment is affected in the form of anterior uveitis or endotheliitis. CMV-associated endotheliitis presents with corneal endothelial damage due to the intense viral load in the aqueous fluid. Clinical findings include local stromal edema, mild anterior chamber reaction, KP, elevated IOP, and stromal iris atrophy. Antiviral agents are used in treatment.^7^

Various cases of CMV endotheliitis after keratoplasty have been reported in the literature.^8,9,10^ In such cases, differential diagnosis of corneal graft rejection and viral infection can be challenging due to the similar clinical findings of both diseases. Furthermore, CMV infection can lead to endothelial inflammation and trigger graft rejection.^11^ Microbiological examination of the aqueous humor is diagnostic for CMV endetheliitis after PPK.^12^ However, in cases where PCR cannot be performed, the typical pattern of KPs may also be helpful for differential diagnosis. Conversely, in immune-related graft rejection, KPs form a line along the edema margins called a “Khodadoust line”. In CMV-related endotheliitis, KPs are usually coin-shaped and located in the center of the edema. Additionally, CMV-associated endotheliitis is often accompanied by anterior uveitis and elevated IOP. Immunosuppressive agents are effective in the treatment of immune-related graft rejection, whereas antiviral therapies such as ganciclovir or foscarnet can provide clinical improvement in CMV-associated endotheliitis.^1^ Our patient developed epithelial defect and corneal edema at postoperative 18 months under topical steroid and systemic cyclosporine treatment, and was diagnosed as graft rejection. At that time, the absence of anterior chamber reaction or typical endothelial plaque, normal IOP, symptom relief after intensive steroid administration without antiviral therapy, and the long-term disease-free period ruled out the diagnosis of CMV endotheliitis. However, at postoperative 4 years, CMV endotheliitis was suspected due to localized stromal edema accompanied by coin-shaped KPs and anterior chamber reaction, and the diagnosis was confirmed by PCR.

The source of CMV infection is uncertain in patients who underwent keratoplasty. It may arise from the donor tissue or be caused by reactivation of a preexisting CMV infection in the host.^1^ Although donors are routinely evaluated for hepatitis virus and human immunodeficiency virus (HIV) serology before corneal transplantation, CMV serology is usually unknown. Therefore, transmission may occur from a CMV-positive donor cornea to a CMV-negative host. However, it was thought to occur rarely.^13^ Additionally, although the mean incubation period for donor-derived viral endotheliitis after corneal transplantation is not certain yet, systemic CMV infection is known to begin within 6 weeks to 6 months.^14^ Similarly, in CMV retinitis the symptoms usually appear early at post-transplantation 9 months.^6^ Therefore, CMV-associated endotheliitis is expected to present clinical signs and symptoms at early post-transplantation period. Another possible mechanism of CMV infection is reactivation of latent CMV virus in the host tissue. The topical or systemic immunosuppressive therapy used after keratoplasty may lead to CMV reactivation and endotheliitis.^1^ In our patient, the preoperative CMV serology was unknown. Still, disease onset at postoperative year 4 and the patient’s long-term use of immunosuppressive therapy suggest CMV reactivation. There was no evidence of retinitis or systemic CMV infection in our patient’s ocular or systemic evaluations.

The ocular response to viral infections is mediated mainly by the cellular immune system. Zheng et al.^15^ first inoculated rabbit eyes with an inactivated HSV, and then infected intracamerally with live HSV. They observed corneal endotheliitis and suspected anterior chamber-associated immune deviation in its pathogenesis. Accordingly, suppression of cellular immunity and T-cell-mediated delayed-type hypersensitivity results in loss of the antiviral protection, and the endothelial cells are damaged by the virus.^15^ Similarly, Koizumi et al.^7^ also reported that anterior chamber-associated immune deviation may play a role in CMV reactivation. The reason for CMV reactivation in the anterior chamber can be local and/or systemic immunosuppressive therapy.^1,6^ Our patient also developed CMV endotheliitis after immunosuppressive therapy. In the literature, topical steroid use has been reported in 96% of patients with CMV endotheliitis, suggesting that local immunosuppression may also trigger CMV reactivation.^1^

In conclusion, CMV-associated corneal endotheliitis can occur in healthy individuals without systemic symptoms due to viral reactivation. In keratouveitis patients with corneal edema, coin-shaped KPs, and elevated IOP, CMV should be considered as a potential cause of viral endotheliitis in addition to HSVs and VZV. The isolation of viral DNA by PCR is very useful in differential diagnosis, and ganciclovir therapy should be initiated without delay.
